# New Drugs for the Treatment of *Pseudomonas aeruginosa* Infections with Limited Treatment Options: A Narrative Review

**DOI:** 10.3390/antibiotics11050579

**Published:** 2022-04-26

**Authors:** Angela Raffaella Losito, Francesca Raffaelli, Paola Del Giacomo, Mario Tumbarello

**Affiliations:** 1 Dipartimento di Scienze di Laboratorio e Infettivologiche, Fondazione Policlinico Universitario A. Gemelli IRCCS, 00168 Rome, Italy; angelaraffaella.losito@policlinicogemelli.it (A.R.L.); francesca.raffaelli@policlinicogemelli.it (F.R.); paola.delgiacomo@policlinicogemelli.it (P.D.G.); 2 Dipartimento di Biotecnologie Mediche, Università degli Studi di Siena, 53100 Siena, Italy; 3 UOC Malattie Infettive e Tropicali, Azienda Ospedaliero Universitaria Senese, 53100 Siena, Italy

**Keywords:** *Pseudomonas aeruginosa*, difficult-to-treat resistant (DTR), new β-lactam–β-lactamase inhibitor combinations, cefiderocol, imipenem-cilastatin-relebactam, meropenem-vaborbactam, plazomicin, fosfomycin combination strategy

## Abstract

*P. aeruginosa* is still one of the most threatening pathogens responsible for serious hospital-acquired infections. It is intrinsically resistant to many antimicrobial agents and additional acquired resistance further complicates the management of such infections. High rates of combined antimicrobial resistance persist in many countries, especially in the eastern and south-eastern parts of Europe. The aim of this narrative review is to provide a comprehensive assessment of the epidemiology, latest data, and clinical evidence on the current and new available drugs active against *P. aeruginosa* isolates with limited treatment options. The latest evidence and recommendations supporting the use of ceftolozane-tazobactam and ceftazidime-avibactam, characterized by targeted clinical activity against a significant proportion of *P. aeruginosa* strains with limited treatment options, are described based on a review of the latest microbiological and clinical studies. Cefiderocol, with excellent in vitro activity against *P. aeruginosa* isolates, good stability to all β-lactamases and against porin and efflux pumps mutations, is also examined. New carbapenem combinations are explored, reviewing the latest experimental and initial clinical evidence. One section is devoted to a review of new anti-pseudomonal antibiotics in the pipeline, such as cefepime-taniborbactam and cefepime-zidebactam. Finally, other “old” antimicrobials, mainly fosfomycin, that can be used as combination strategies, are described.

## 1. Introduction

*Pseudomonas aeruginosa* is one of the most threatening pathogens, especially in healthcare settings and in immunocompromised patients due to both its extraordinary capability to develop additional in vivo resistance to different antibiotics and to its virulence. Various molecular mechanisms, intrinsic, acquired, and adaptive, are responsible for *P. aeruginosa* antimicrobial resistance. Notably, in one clinical isolate, different mechanisms can be often simultaneously present. Although each of them is related to a specific class of antibiotics, multiple mechanisms mediate variable levels of resistance to each class of antibiotics. Deficiency of OprD and overproduction of active efflux pumps, AmpC β-lactamase, extended-spectrum β-lactamases (ESBL), and carbapenemases, especially metallo-β-lactamase (MBL) production, have been reported as the main contributors to multi-drug resistant phenotypes of *P. aeruginosa* isolates [1]. The contribution of each mechanism varies widely by geographic area. According to the established definitions, multidrug-resistant (MDR) *P. aeruginosa* is non-susceptible to at least one agent in three or more antimicrobial categories, while extensively drug-resistant (XDR) *P. aeruginosa* is non-susceptible to all but two or fewer antibiotic classes among anti-pseudomonal cephalosporins, anti-pseudomonal penicillins plus β-lactamase inhibitors, monobactams, anti-pseudomonal carbapenems, aminoglycosides, fluoroquinolones, phosphonic acid and polymyxins [2].

Such definitions were adopted before the introduction of the novel β-lactam–β-lactamase inhibitor (BL-BLI) combinations ceftolozane-tazobactam and ceftazidime-avibactam. Thus, more recently a new definition for *P. aeruginosa* strains with resistance to piperacillin-tazobactam, ceftazidime, cefepime, aztreonam, meropenem, imipenem-cilastatin, ciprofloxacin, and levofloxacin but with preserved susceptibility to the novel BL-BLI combinations and colistin was proposed and denominated Difficult-to-Treat (DTR) Resistant *P. aeruginosa* [3].

*P. aeruginosa* has a marked plasticity and is distinguished by its large genome which includes a conserved core genome, and several sets of rare genes and gene islands. This latter group of genes is responsible for the versatility of this pathogen.

Virulence, a strategy that allows *P. aeruginosa* to evade the host immune defense, particularly at the early stages of colonization and acute infection, resides in cell-mediated virulence factors (which are constitutive) but, even more, in the production of secreted virulence factors, largely dependent on the environmental factors and the niche surrounding *P. aeruginosa*. The first, such as lectins, mediate bacterial cells adherence, whereas pili and flagella enable *P. aeruginosa* to move from one niche to another and thus to be present in a wide range of different habitats. Secreted virulence factors (i.e., exotoxins, proteases and other enzymes, pigments, siderophores, and other inorganic compounds with protective function against damage caused by reactive oxygen species) are relevant in the later stages of the infection and invasion, during which bacterial cells proliferate and following damage occurs at the site of infection [1,4]. Formation of biofilms by *P. aeruginosa* is instead the hallmark of chronic infections and indicative of disease progression and long-term persistence [1].

*P. aeruginosa* is a common cause of severe healthcare-associated invasive infections especially pneumonia, bloodstream infections (BSIs), and complicated urinary tract infections (cUTIs). Thus, World Health Organization (WHO) has designated carbapenem-resistant *P. aeruginosa* (CRPA), as one of the priority pathogens for research and development of new antibiotics [5]. 

## 2. Epidemiology

*P. aeruginosa* remains one of the major causes of healthcare-associated infection in Europe [6]. All healthcare institutions have reported *P. aeruginosa* outbreaks and intrahospital infections, as these bacteria can survive on abiotic and biotic surfaces such as medical equipment, resisting disinfection methods, and they can also transiently colonize the intestinal tract while being transmissible from patient to patient, especially among immunocompromised patients and other fragile hosts [4,7].

According to the European Antimicrobial Resistance Surveillance Network (EARS-Net) data, in 2020 in the European Union/European Economic Area (EU/EEA), 30.1% of the *P. aeruginosa* isolates reported were resistant to at least one of the antimicrobial groups under surveillance (piperacillin-tazobactam, fluoroquinolones, ceftazidime, aminoglycosides, and carbapenems). The highest EU/EEA population-weighted mean resistance percentage in 2020 was reported for fluoroquinolones (19.6%), followed by piperacillin-tazobactam (18.8%). For carbapenems, resistance was reported as 17.8% [8]. 

Antimicrobial resistance, particularly carbapenem resistance in *P. aeruginosa*, poses a global therapeutic challenge highlighting the versatility of this pathogen in acquiring and disseminating enzymatic and nonenzymatic resistance mechanisms.

**Figure 1:**
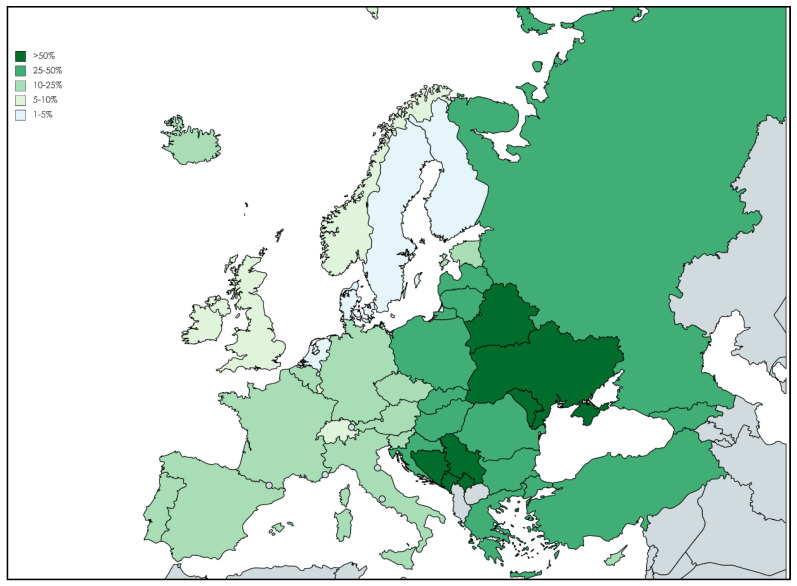
Percentage of invasive *P. aeruginosa* isolates with resistance to carbapenems, 2020. Map was created through the online tool Mapchart.net (https://mapchart.net/world.html, accessed on 20 April 2022).

Wide variability is seen in the proportions of CRPA within the WHO European Region (Figure 1). In 2020, antimicrobial-resistant percentages of below 5% were observed in four (10%) of 41 countries/areas reporting data on this microorganism (Denmark, Finland, the Netherlands, and Sweden), whereas six (15%) countries reported percentages equal to or above 50% (Belarus, Bosnia and Herzegovina, Montenegro, the Republic of Moldova, Serbia and Ukraine). Resistance to five antibiotic classes (piperacillin-tazobactam + fluoroquinolones + ceftazidime + aminoglycosides + carbapenems) is 3.1% [8].

*P. aeruginosa* global clones associated with MDR and XDR phenotypes, so-called high-risk clones, are a growing threat in hospitals worldwide. According to their prevalence, global spread, and association with MDR/XDR profiles and regarding ESBLs and carbapenemases, the worldwide top 10 *P. aeruginosa* high-risk clones include ST235, ST111, ST233, ST244, ST357, ST308, ST175, ST277, ST654, and ST298. Some of them, such as ST357, ST308, and ST298, are also potentially associated with higher virulence [9].

Most *P. aeruginosa* infections with limited treatment options are often reported in Intensive Care Units (ICUs) and in long-term acute care hospitals probably due to the extensive use of antimicrobials, which allows for the selection of this microorganism [10]. A recent analysis based on the EARS-Net data highlighted that countries reporting high proportions of *P. aeruginosa* BSIs of the total reported BSIs were those countries where the rate of acquired resistance in Gram-negative bacteria (GNB) was also generally highest [11]. This finding is probably attributed to shared risk factors such as broad-spectrum antimicrobials consumption [12]. 

In addition, it has been widely reported that in the COVID-19 era, *P. aeruginosa* with documented resistance to multiple antibiotics is a common cause of severe superinfections (i.e., ventilator-associated pneumonia, VAP, and BSIs) among critically ill patients with COVID-19 [13].

Risk factors for acquiring MDR/XDR-*P. aeruginosa* infections include immunodeficiencies, chronic pulmonary diseases (i.e., cystic fibrosis), admission to an ICU in the previous year, and carbapenem or fluoroquinolone-based therapy within the previous 3 months [14]. 

*P. aeruginosa* represents one of the six leading pathogens for deaths associated with resistance [15]. Together with third-generation cephalosporin-resistant *Escherichia coli* and *Klebsiella pneumoniae*, Methicillin-resistant *Staphylococcus aureus* (MRSA), CRPA-related infections caused 67.9% of the total disability-adjusted life years (DALYs) per 100,000 in European Union and European Economic Area in 2015 [16].

Further, hospital length of stay (LOS), readmission rates, and cost per infection are are higher for cases with MDR-*P. aeruginosa* infections relative to those with non-MDR-*P. aeruginosa* infections [17]. 

## 3. Ceftolozane-Tazobactam

Ceftolozane-tazobactam is an expanded-spectrum cephalosporin of fifth generation, combined with a well-known β-lactamase inhibitor. This combination is characterized by enhanced activity against *P. aeruginosa*, including MDR and XDR strains, because of inhibition of its key penicillin-binding proteins (PBPs), and also has high activity, mostly against *Enterobacterales* including ESBL strains [18]. Its novel 3D structure confers better stability to the hydrolysis also due to AmpC β-lactamase, compared with other cephalosporins [19,20], even if AmpC mediated pathways have recently been highlighted to play a part in the emergence of *P. aeruginosa* resistance strains to ceftolozane-tazobactam [21], also after treatment [22]. The development of in vivo resistance to ceftolozane-tazobactam has been identified in *P. aeruginosa* isolates due to the selection and emergence of acquired extended-spectrum variants in class D β-lactamases and oxacillinases (OXA), which hydrolyze ceftolozane and are not efficaciously tazobactam inhibited, as previously outlined for OXA-2 [23,24] and OXA-10 [25]. Porin permeability changes and hyperexpression of efflux pumps were not expected to impact susceptibility [26], even if, already in 2017, data from BSAC bacteremia surveillance showed resistance patterns in cases of increased efflux [27], and a more recent paper reports the emergence of a complex resistance picture, following ceftolozane-tazobactam therapy, mediated, among others, also by OprD porin mutation and upregulation of efflux pumps [28]. Ceftolozane-tazobactam is not active in carbapenemase-producing strains, limiting therapeutic options in *P. aeruginosa* resistant to carbapenems, and in particular, production of MBL has been involved in the detection of ceftolozane-tazobactam nonsusceptible *P. aeruginosa* strains [9,27]. Ceftolozane-tazobactam has also been shown to not explicate marked activity in the case of *P. aeruginosa* under biofilm state in vitro [29], as was also confirmed in a pharmacodynamic model simulating foreign-body infections [30]. 

Phase 3 trials showed good efficacy and safety for ceftolozane-tazobactam treatment, including in the setting of MDR *P. aeruginosa* in complicated urinary tract infections where 2.9% of uropathogens at baseline was represented by *P. aeruginosa* [31], in complicated intra-abdominal infections combined with metronidazole where *P. aeruginosa* was isolated from intra-abdominal specimens at a baseline of 8.9% in the microbiological intent-to-treat (MITT) population [32], and at high dose in nosocomial pneumoniae, where *P. aeruginosa* made up the 25% of MITT population [33]. Thus, approval for the treatment of these clinical pictures was received from the Food and Drug Administration (FDA) and European Medicines Agency (EMA). In the recent antimicrobial-resistant treatment guidance, the use of ceftolozane-tazobactam has been recommended among the preferred options for DTR *P. aeruginosa* infections because of generally high susceptibility rates over the other mentioned alternatives in any clinical context; moreover, the guidance suggests a high dose schedule outside uncomplicated UTI [34], which is notably effective when promptly administered [35] and has a better safety profile [36]. European Society of Clinical Microbiology and Infectious Diseases (ESCMID) guidelines, in the context of severe infections caused by CRPA, suggest the treatment with ceftolozane-tazobactam if in vitro susceptible [37], due to the significant benefit of ceftolozane-tazobactam versus polymyxin or aminoglycoside-based combination treatment regimens, in term of clinical cure to which ceftolozane-tazobactam treatment was indipendently associated [36].

A recent European survey, part of the Program to Assess Ceftolozane/Tazobactam Susceptibility (PACTS), monitoring in vitro activity of ceftolozane-tazobactam found 94.1% of all *P. aeruginosa* isolates in Western Europe were susceptible and 80.9% in Eastern Europe (better rates seen only in colistin); but there were lower susceptibility rates ( 75.2% and 59.2% in Western and Eastern Europe, respectively) if CRPA isolates were taken into account [38]. Similar trends have been observed in real-life multicenter studies where susceptibility of *P. aeruginosa* isolates to ceftolozane-tazobactam was 95.7% for cUTI and 85.3% for cIAI in the context of Gram-negative intra-abdominal and urinary infections, of which 16.7% were due to *P. aeruginosa* strains, occurring in Spanish intensive care units [39]. In addition, there was 88.7% susceptibility to ceftolozane-tazobactam in a partial cohort tested (71.2%) of *P. aeruginosa* isolates (95.8% MDR and 37.7% XDR) that represented 91.1% of the entire cohort of MDR Gram-negative infections analyzed in US medical centers [40]. Lower ceftolozane-tazobactam susceptibility rates have been reported in MDR and XDR *P. aeruginosa* strains worldwide [41], as confirmed in accurate European data distributions of resistant phenotypes which reports up to 48% ceftolozane-tazobactam susceptibility in combined β-lactam-resistant (piperacillin-tazobactam, meropenem, imipenem, and ceftazidime) *P. aeruginosa* isolates [42]. 

Efficacy data based on real-world experience was evaluated among patients with MDR *P. aeruginosa* infections in eight U.S. medical centers; clinical failure and 30-day mortality occurred in 37.6% and 17.3% of patients, respectively, and new ceftolozane-tazobactam resistance in *P. aeruginosa* MDR isolates was detected in 9.7% of cases, although follow-up cultures were available in only one-fifth of cases [40].

A large clinical experience of ceftolozane-tazobactam treatment exclusively in various types of *P. aeruginosa* infections, of which 50.5% of strains were XDR and 78.2% were resistant to at least one carbapenem, resulted in an overall clinical success of 83.2%, but lower rates were observed in patients with sepsis or undergoing continuous renal replacement therapy (CRRT) [43]. 

In a recent multicenter retrospective cohort of critically ill ICU patients affected by severe infections due to *P. aeruginosa* with different resistance patterns and 83.3% carbapenem-resistant (XDR 48.4% and MDR 36.8%), a beneficial clinical response was observed in 71.6% of patients, with a microbiological eradication rate of 42.1% and no outcome differences in the case of combination therapy [44].

Furthermore, in the specific context of hematologic malignancy patients with *P. aeruginosa* infections, ceftolozane-tazobactam has been found to be as effective as other treatment options, including in those infections caused by XDR strains [45].

In brief, ceftolozane-tazobactam represents a good option for the treatment of susceptible MDR/XDR *P. aeruginosa* infections, representing a first-line option in the CRPA as recently assessed by European guidelines [36], and also in the context of ICU severe infections and complex clinical scenarios such as real-life experiences assessed in studies [44,45]. Caution should be advised in the determination of optimal dosing, e.g., in the presence of renal impairment [43], in appropriate dosing to achieve infusion appropriateness [46], and in possible combination therapy in selected settings such as high-inoculum infections where the emergence of resistance may be realized [47].

## 4. Ceftazidime-Avibactam

Ceftazidime-avibactam is a novel combination of a well-known antipseudomonal third-generation cephalosporin with a new (non-β-lactam) β-lactamase inhibitor. This new compound acts through ceftazidime, which carries out its activity by linking to PBPs of the Gram-negative aerobic pathogens and *P. aeruginosa* walls, including MDR or XDR strains, thanks to avibactam’s ability to overcome β-lactamases Ambler class type A (ESBL, *Klebsiella pneumoniae* carbapenemases KPC), C (AmpC cephalosporinases) and partially class D carbapenemases such as OXA-48 in *K. pneumoniae*. It does not retain activity against metallo-β-lactamases [48,49]. 

Use of ceftazidime-avibactam, also among patients with *P. aeruginosa* infections, has been extensively investigated in phase 3 trials. High levels of efficacy and safety were observed in a RECAPTURE trial for treatment of cUTI where the *P. aeruginosa* isolates rate was 4.7%, representing the most frequent isolate among non-*Enterobacterales*’ [50], in the cUTI and cIAI of the REPRISE trial where it accounted for almost 7% of all isolates [51], in cIAI cohorts of RECLAIM studies where *P. aeruginosa* strains accounted for more than 8% and 12.5%, respectively [52,53], and in hospital-acquired pneumonia (HAP) including VAP, where *P. aeruginosa* was one of two predominant isolates (30% of microbiologically modified intention-to-treat population) [54]. In a subsequent pooled analysis from all the above mentioned clinical trials, and also in MDR isolates which accounted for 34.9% of the pooled *P. aeruginosa* dataset, similar microbiological and clinical responses to comparators were noted [55]. 

Recent IDSA treatment guidelines for Gram-negative bacterial antimicrobial-resistant infections suggest ceftazidime-avibactam therapy in the settings of virtually all DTR *P. aeruginosa* infections with limited therapeutic options [34]. According to ESCMID guidelines, there is a lack of evidence to suggest ceftazidime-avibactam for the treatment of serious infections due to CRPA [37].

Real-life experiences on MDR *P. aeruginosa* treatment suggested encouraging levels of effectiveness; firstly, in a cohort of complex medical conditions patients with high-severity index MDR Gram-negative infections, 31% of which were due to *P. aeruginosa*, mostly carbapenem-resistant [56], and secondly as a valid treatment alternative in a retrospective cohort study on patients with of MDR/XDR *P. aeruginosa* infections (61 first episodes), although not so promptly treated [57]. In addition, high rates of clinical cure (87.8%) were observed in serious infections caused by MDR and XDR *P. aeruginosa* isolates other than carbapenem-resistant within a patient cohort with Gram-negative infections due to non-carbapenem-resistant *Enterobacterales* (CRE) MDR bacteria (33/41; 80.5% *P. aeruginosa* infections) [58]. A recent review of the actual practice of ceftazidime-avibactam treatment of infections with limited options made up of 1718 patients with *Enterobacterales* including carbapenemase-producing *Enterobacterales* (CPE) and CRE strains and 150 patients with *P. aeruginosa* including carbapenem-resistant, MDR, and XDR strains, presents high-quality data on the favorable use of this compound, also in the setting of MDR *P. aeruginosa* infections [59].

According to the INFORM database, the susceptibility of *P. aeruginosa* to ceftazidime-avibactam has been reported in a range from 88.7% to 93.2% in the four main geographical areas, although these rates were lower in the presence of concomitant resistance to β-lactams or meropenem [60]. Considering local distributions in Western European countries, compared with newer BL-BLI antipseudomonal compounds, ceftazidime-avibactam retains the best activity to different single phenotypic *P. aeruginosa* resistance patterns such as piperacillin-tazobactam-resistant (91.2%), meropenem-resistant (81.6%), imipenem-resistant (92.6%), ceftazidime-resistant (87.8%) [42]. 

In a comparative analysis of BL-BLI against *P. aeruginosa* from patients hospitalized with pneumonia in 2020 in Europe, after colistin, ceftazidime-avibactam was the most active against resistant subsets from Western Europe (from 92.6% susceptibility when tested against imipenem-resistant isolates to 87.8% against ceftazidime-resistant strains) [42].

In DTR *P. aeruginosa* such as in combined β-lactam-resistant strains, lower ceftazidime-avibactam susceptibility rates equal to 64% have been reported among European pneumonia infections [42]. In the setting of clinical respiratory *P. aeruginosa* isolates, among carbapenemase-producing strains that are MBL negative, a susceptibility of 71.7% to ceftazidime-avibactam has been reported, a percentage that decreases to under 19.1% in MBL positivity [61].

Resistance to ceftazidime-avibactam in *P. aeruginosa* isolates has been also observed due to porin or efflux pumps modifications, recently also in the context of previous antibiotic treatment [28,62,63]. Resistance rates of 37.5% to ceftazidime-avibactam have been described in cystic fibrosis patients harboring piperacillin-tazobactam resistant *P. aeruginosa* isolates, due to OprD mutations [64]. AmpC mutations, emerging after MDR *P. aeruginosa* infections treatment with ceftolozane-tazobactam and responsible for resistance to it, are also involved in ceftazidime-avibactam cross-resistance [22]. Emerging acquired mutations in OXA-2 and OXA-10 have been reported to be responsible for in vivo resistance to ceftazidime-avibactam in *P. aeruginosa* and also as cross resistance to ceftolozane-tazobactam [24,25,65].

It should be considered that microbiological failure and emergence of ceftazidime-avibactam resistance have been associated with *P. aeruginosa* infection in a cohort of critically ill patients with Gram-negative infections experiencing continuous infusion [66].

Mixed data have been observed on its use in combination in *P. aeruginosa* infections, ranging from emerging as a predictor of response [57] to not being associated with less clinical failure and mortality but with greater adverse renal effects [56].

In conclusion, from reviewed data sourced from clinical real-life experiences, ceftazidime-avibactam emerges as a good option for the treatment of MDR/XDR *P. aeruginosa* infections, also in the case of strains harboring carbapenemases, and also in complex clinical conditions [56]. Indeed, it is suggested as a targeted treatment in DTR *P. aeruginosa* infections with limited therapeutic options [34], thanks to high susceptibility rates in such cases. On the other hand, serious infections should be treated with caution in terms of cure and microbiological failure [37,66]

## 5. Cefiderocol

**Figure 2:**
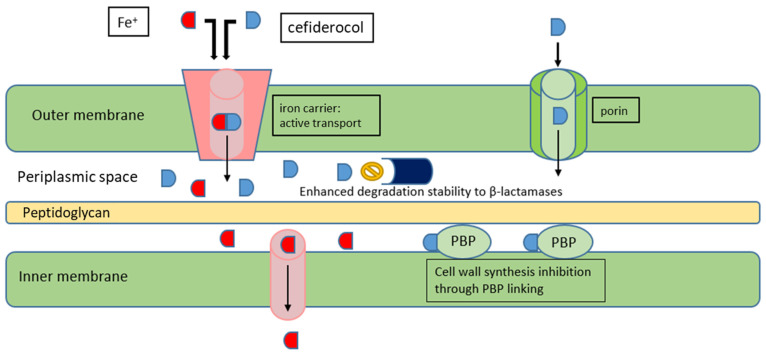
Mechanism of action of cefiderocol.

Cefiderocol is a siderophore cephalosporin with activity against a wide spectrum of Gram-negative micro-organisms, including resistant ones. It performs its peculiar penetration activity by linking to ferric iron, which allows it to use active iron carriers to permeate the bacterial outer membrane (Figure 2). This novel mechanism, together with its high stability against all β-lactamases, including carbapenemases, MBLs, and AmpC, and against porin and efflux pumps mutations, accounts for its broad activity [47,67].

In a 2014–2016 wide collection from 52 countries of difficult to treat Gram-negative isolates, cefiderocol showed a potent in vitro activity, with 99.2% susceptibility for MDR *P. aeruginosa* and also maintaining 99% and 98.8% susceptibility, respectively, in the context of ceftolozane-tazobactam and ceftazidime-avibactam resistant isolates [68]. 

*P. aeruginosa* data from the latest SENTRY Antimicrobial Surveillance Program on the in vitro activity of cefiderocol in Gram-negative US and European isolates showed that cefiderocol achieved 99.6% susceptibility against all isolates according to CLSI criteria, and slightly lower (97.3%) when dealing with XDR isolates but still far superior to the newer BL-BLI combinations (imipenem-relebactam 73.0%, ceftazidime-avibactam 73.4%, and ceftolozane-tazobactam 72.3%) as cefiderocol also shows activity as a strong inhibitor of BL-BLI-resistant *P. aeruginosa*. Cefiderocol retained complete susceptibility in imipenem-relebactam-resistant isolates and remarkable susceptibility rates for isolates resistant to ceftazidime-avibactam and ceftolozane-tazobactam (91.6% and 88.3%, respectively), as well as for combined resistance to all three new compounds (100.0%) [69].

The use of cefiderocol, its efficacy, and safety, also among patients with *P. aeruginosa* infections, has been investigated in phase 3 trials. The CREDIBLE-CR trial assessed its efficacy in carbapenem-resistant Gram-negative infections (nosocomial pneumoniae, bloodstream infections and sepsis, cUTIs), where *P. aeruginosa* infections accounted for 19% of the MITT population, and 15% of all pathogens treated in the cefiderocol arm were represented by *P. aeruginosa*. Similar performance in terms of clinical and microbiological efficacy compared with the best available therapy (BAT) emerged. It is noteworthy that the highest all-cause mortality was described in the cefiderocol arm (25% vs 11%), but after stratification of the data, all-cause mortality in monomicrobial *Acinetobacter spp*. infections was 50% vs 18% in *P. aeruginosa* infections, where the same mortality rate was recorded in the two treatment arms and there was no difference in clinical cure and microbiological persistence [70].

In the APEKS-NP trial, which assessed all-cause mortality at day 14 in nosocomial pneumonia caused by GNB including MDR strains, 16% of all baseline pathogens were represented by *P. aeruginosa*, 8% of which were carbapenemase producers, cefiderocol showed non-inferiority to high-dose extended-infusion meropenem, and similar tolerability [71].

In a recent evaluation of the efficacy of the new drug against MBL-producing pathogens across the two trials (19.5% in CREDIBLE-CR and 3.8% in APEKS-NP), in which almost one third of MBL strains overall consisted of *P. aeruginosa*, cefiderocol monotherapy showed higher rates of clinical cure and microbiological eradication than comparators, providing a benefit in MBL-producing CRPA infections. Among non-fermenters, MBLs were mainly represented by IMP, NDM, and VIM enzymes [72]. 

A post hoc analysis in BSIs caused by GNB across phase 2 and phase 3 randomized clinical studies assessed treatment with cefiderocol as a valuable option because of high bacterial eradication in this clinical picture, also in the setting of carbapenem-resistant strains, even if data on *P. aeruginosa* infections were exiguous as it was isolated in less than 5% of bacteremia [73].

According to IDSA guidelines, cefiderocol is included among the recommended treatment options for uncomplicated cystitis, pyelonephritis, and cUTIs due to DTR *P. aeruginosa*, and as an alternative therapy for infections outside the urinary tract if first-line agents are unavailable or not tolerated [34]. Poor evidence was available from the CREDIBLE-CR trial according to ESCMID guidelines to recommend cefiderocol in the treatment of CRPA infections [37].

A short report from real-life clinical experience reported the successful use of cefiderocol in a case series of three patients, one of whom was affected by a polymicrobial infection with an MBL *P. aeruginosa* isolate treated in combination, and reviewed other previously described single cases, including some due to XDR *P. aeruginosa* strains, who recovered [74]. Regarding real hands-on practice, a clinical cure and microbiological cure rates of 70.6% and 76.5%, respectively, have been recently described among a real-life compassionate experience with cefiderocol in the treatment of 17 miscellaneous infections caused by XDR and difficult to treat resistant *P. aeruginosa* with no further possible therapeutic options, mostly treated in combined regimens [75]. In another real-life clinical setting using cefiderocol in combination as a salvage treatment in 13 difficult-to-treat infections caused by XDR GNB, 15% of which were XDR *P. aeruginosa*, in challenging clinical situations such as immunocompromised or critically ill patients or in surgical infections with prior treatment failure, overall microbiological clearance was attained with a nearly 77% 30-day survival rate [76].

Cefiderocol-resistant *P. aeruginosa* isolates at the treatment baseline have been described that are probably due to adjunctive mechanisms other than carbapenemases [77], which may be involved in vitro [78]. However, a *P. aeruginosa* sub-strain which is non-susceptible to cefiderocol due to mutations in iron transport pathways was isolated from an experienced patient without prior cefiderocol exposure [79]; therefore sensitivity has to be tested. Most papers report on cefiderocol combination regimens [75,76,80] rather than than monotherapy [81], but further studies are needed to better assess any possible outcome impact.

In summary cefiderocol, because of its strong activity and the high susceptibility of DTR *P. aeruginosa* strains, which is even higher than the newer BL-BLI combinations [69], shows considerable potential for the treatment of related infections. Very recent data found that cefiderocol, due to high microbiological eradication and clinical cure rates [72,73,75], could represent an important therapeutic option in DTR *P. aeruginosa* infections [34], in particular in the context of XDR and carbapenem-resistant strains, above all for MBL producers, and also in difficult clinical pictures [76]. Testing the sensitivity of the compound is anyway suggested since resistant *P. aeruginosa* isolates have been described. Further data are needed to assess the impact of its use in combination.

## 6. Imipenem-Cilastatin-Relebactam

Imipenem-cilastatin-relebactam is a new antibiotic combination consisting of a carbapenem, imipenem, and a potent non-β-lactam bicyclic diazabicyclooctane β-lactamase inhibitor, relebactam, structurally similar to avibactam with an additional piperidine ring.

Imipenem-cilastatin-relebactam is active against class A β-lactamases, which include ESBLs and KPCs, and class C β-lactamases (AmpCs). The addition of relebactam does not improve the activity of imipenem against OXA-48 and Ambler class B MBLs (IMP, VIM, and NDM) producing isolates [49,82].

The main imipenem resistance mechanisms in *P. aeruginosa* are loss of the outer membrane entry porin OprD and high-level expression of the chromosomally-encoded AmpC enzyme [83].

Imipenem-cilastatin-relebactam is active against carbapenem-resistant strains with an impermeability resistance mechanism since neither imipenem nor relebactam is a substrate of the most common multidrug efflux pumps (MexA-MexB-OprM) [84,85]. The inhibition of the chromosomal AmpC enzyme by relebactam restores susceptibility to many MDR isolates of *P. aeruginosa* by its capacity to inhibit the low-level hydrolysis of imipenem by AmpC and the characteristic of not inducing the production of AmpC [86], including those with over-expression of efflux pumps [83]. Indeed, in *P. aeruginosa* isolates with OprD-deficiency, imipenem-cilastatin-relebactam was active with MIC of imipenem decreased fourfold (from 16 to 64 mg/lt to 1 to 4 mg/lt) [84,87].

Relebactam restored imipenem susceptibility to 75–92% of imipenem non-susceptible isolates [88,89,90,91,92].

Data from the SMART (Study for Monitoring Antimicrobial Resistance Trends) surveillance program of imipenem-resistant *P. aeruginosa* in the USA showed that relebactam improved the activity of imipenem in 80.5% of isolates [93]. In particular, imipenem-cilastatin-relebactam retained in vitro activity against 82.2% of MDR-*P. aeruginosa* isolates and 62.2% of DTR-*P. aeruginosa* isolates [94].

Focusing on *P. aeruginosa* isolates from intra-abdominal infections and from the urinary tract, the susceptibility to imipenem-cilastatin-relebactam was 96.7% and 96.4%, respectively, and imipenem-nonsusceptible and MDR-*P. aeruginosa* strains were observed to have 85% and 87.3% susceptibility, respectively [95]. These data are consistent with those collected in a Canadian study that revealed that imipenem-cilastatin-relebactam in vitro activity was 70.8% against MDR-*P. aeruginosa* isolates [96].

In the SMART European surveillance study, data from the period between 2015 and 2017 showed that among *P. aeruginosa*, 94.4% of IAI and 93% of UTI isolates were susceptible to imipenem-cilastatin-relebactam, as were 74.4% of imipenem-nonsusceptible and 79.8% of MDR isolates from IAIs and UTIs combined [85]. Focusing on patients with respiratory tract infections in an ICU setting, data from SMART US between 2017 and 2019 showed that imipenem-cilastatin-relebactam maintained activity against 91% of *P. aeruginosa* isolates from ICU patients, which is consistent with a previous study, and 66% of MDR-*P. aeruginosa* from ICUs. Furthermore, the activity of imipenem-cilastatin-relebactam was slightly lower than that of ceftolozane-tazobactam, but imipenem-cilastatin-relebactam maintained activity against 58% of ceftolozane-tazobactam-nonsusceptible isolates [97], mainly due to AmpC mutations [22]. Indeed, a recent study showed that imipenem-cilastatin-relebactam stepwise resistance development was not facilitated in clinical XDR strains that had already acquired ceftolozane-tazobactam resistance during treatment and was not significantly increased for the tested XDR high-risk clone isolates [98].

In pneumonia from Eastern European and Mediterranean regions, imipenem-cilastatin-relebactam was in vitro slightly more active than the other BL-BLIs combinations, with susceptibility rates ranging from 81.4% for imipenem-resistant isolates to 64.5% for meropenem-resistant strains [42].

In a study assessing the development of resistance during exposure to imipenem-cilastatin-relebactam using in vitro simulations, an increase in MIC and bacterial regrowth in the 14-day model were observed for *P. aeruginosa*. The development of resistance was prevented with the addition of amikacin [99]. 

The first in vivo studies using a human-simulated regimen demonstrated that imipenem-cilastatin-relebactam therapy was superior compared to imipenem regimens against MDR-*P. aeruginosa* over a wide range of imipenem-cilastatin-relebactam MICs [100].

Preclinical analyses, lung penetration studies, population PK modeling, and probability of target attainment simulations all further support the 1.25 g dose (500 mg imipenem, 500 mg cilastatin, and 250 mg relebactam) infused over 30 min every 6 h and appropriately adjusted for renal function [101].

Imipenem-cilastatin-relebactam was approved by FDA in 2019 for the treatment of cUTI, including pyelonephritis, and cIAI in adult patients [102], and in 2020, it was approved by the EMA for the treatment of infections caused by aerobic GNB in adults with limited treatment options [103].

Clinical data for the use of imipenem-cilastatin-relebactam were evaluated in phase 3 multicenter, randomized, double-blind clinical trials. The RESTORE IMI-1 compared imipenem-cilastatin-relebactam with imipenem and colistin for the treatment of imipenem-non-susceptible bacterial infections (including HAP/VAP, cIAI, or cUTI). *P. aeruginosa* was the most commonly isolated organism (36/47, 77%), including all HAP/VAP patients and all but one cIAI patient. The overall favorable response was similar among the two groups (71.4% for imipenem-cilastatin-relebactam vs. 70.0% for colistin + imipenem-cilastatin). The overall favorable clinical response was higher with imipenem-cilastatin-relebactam among the subset of patients with a *P. aeruginosa* infection (81% vs. 63%), although this was not statistically significant. The twenty-eight-day all-cause mortality was lower with imipenem-cilastatin-relebactam (9.5% vs 30%), and the clinical response at 28 days was significantly higher with imipenem-cilastatin-relebactam (71% vs 40%) [104].

The RESTORE IMI-2 trial compared imipenem-cilastatin-relebactam with piperacillin-tazobactam, with empiric linezolid administered in both treatment arms, for the treatment of HAP/VAP. *P. aeruginosa* was isolated in 18.9% (82/531) of patients. Imipenem-cilastatin-relebactam was noninferior to piperacillin-tazobactam for the 28-day all-cause mortality (15.9% vs. 21.3%). Patients with *P. aeruginosa* infections had comparable microbiological eradication rates in both treatment arms (67% imipenem-cilastatin-relebactam vs. 72% piperacillin-tazobactam), but lower clinical response and higher day 28 mortality rates in the imipenem-cilastatin-relebactam arm; this may be attributable, according to the authors, to differences between the treatment arms unrelated to the causative pathogen [101].

Rebold et al. recently published a real-life study on 21 patients with mixed infection (52% LTRI) caused by various pathogens, mainly *P. aeruginosa* (16/21, 76%), nearly all MDR (15/16, 94%), treated with imipenem-cilastatin-relebactam. The mortality rate was 33% and clinical cure occurred in 62% of patients. Microbiological recurrence and subsequent cultures occurred in 5/21 patients. Two of these were isolates with increased imipenem-cilastatin-relebactam MICs relative to the index culture, from 1.5/4 and 2/4 mg/L (susceptible) to 12/4 and 8/4 mg/L (resistant) [105].

IDSA guidance on the treatment of *P. aeruginosa* with difficult-to-treat resistance suggests imipenem-cilastatin-relebactam therapy for cystitis, pyelonephritis, or cUTI and also for infections outside of the urinary tract [34]. Instead, given the paucity of data on CRPA, ESCMID guidelines conclude on very low-certainty evidence for non-inferiority of imipenem-relebactam compared with colistin-meropenem combination therapy [37]. Given the activity of imipenem-cilastatin-relebactam against ceftolozane-tazobactam-nonsusceptible isolates due to AmpC mutations [22,97], it could be considered a reasonable treatment option in these resistant strains. Data available about the efficacy of imipenem-cilastatin-relebactam are derived mainly from in vitro studies; therefore, future studies are needed to define its role in clinical practice, including the potential to develop resistance on treatment.

## 7. Meropenem-Vaborbactam

Meropenem-vaborbactam is an antimicrobial combination of a well-known, broad spectrum carbapenem and a novel cyclic boronic acid β-lactamase inhibitor with a high affinity of serine residues which enables it to perform as a competitive inhibitor by forming a covalent bond with the β-lactamase without undergoing hydrolysis [106].

Meropenem-vaborbactam is active against Ambler class A and C β-lactamase with an excellent in vitro activity against KPC but is not active against MBLs or oxacillinases with carbapenemase activity [106].

The activity of meropenem-vaborbactam against *P. aeruginosa* strains was found to be overall similar to that of meropenem alone. In a study conducted in the US, Lapuebla et al. showed that 79% of *P. aeruginosa* isolates were susceptible to meropenem, and the rate was not modified by adding vaborbactam [107]. This is apparently because meropenem resistance in *P. aeruginosa* is primarily due to porin mutations or upregulation of efflux pumps, mechanisms that are not antagonized by vaborbactam [108].

However, another study demonstrated that, with some *P. aeruginosa* strains, the addition of vaborbactam produced an increased bacterial killing in a neutropenic mouse thigh infection model, despite the in vitro MIC being the same for both agents, suggesting that these strains may contain an inducible β-lactamase that is inhibited by vaborbactam [109].

In a recently published study assessing the activity of meropenem-vaborbactam for the treatment of pneumonia caused by *P. aeruginosa* (3.193 isolates) and *Enterobacterales* (4.790 isolates) between 2014 and 2018 from patients in US hospitals, 89.5% of *P. aeruginosa* were susceptible to meropenem-vaborbactam, among these the susceptibility rates for MDR (21.8%) and XDR (13.8%) were 59.0% and 48.6%, respectively [110].

Data from the SENTRY Antimicrobial Surveillance Program (2014–2019) showed that the in vitro meropenem-vaborbactam susceptibility of *P. aeruginosa* strains from patients with HAP and VAP in European hospitals was 82.1% overall. In Western Europe, the sensitivity rate was higher (89.7%), mainly due to the greater spread of KPC in this area, whereas in Eastern Europe, MBL and OXA carbapenemase, against which meropenem-vaborbactam is inactive, are more common. The susceptibility to meropenem-vaborbactam in MDR *P. aeruginosa* (27%) was 41% and susceptibility to meropenem alone was 13%. The CRPA strains were not genetically characterized. In ICU patients, meropenem-vaborbactam was active against 73.2% of *P. aeruginosa* isolates, of which 57% were sensitive to meropenem [111]. 

A recent study assessing the in vitro activity of the newer BL-BLI against *P. aeruginosa* isolates from patients with pneumonia in Europe in 2020 found that susceptibility rates to meropenem-vaborbactam were lower, especially among resistant strains. Indeed, the overall susceptibility to meropenem-vaborbactam was 88.7%, which was reduced to 5.7% and 4.0% against meropenem and β-lactam-resistant (piperacillin-tazobactam, meropenem, imipenem, ceftazidime) isolates, respectively [42].

Ultimately, even if vaborbactam is not expected to increase the coverage of meropenem on MDR *P. aeruginosa*, studies have demonstrated in vitro activity of meropenem-vaborbactam [111] against MDR and XDR strains, which occur at higher rates in Western Europe, maybe reflecting the greater spread of KPC in this area, against which meropenem-vaborbactam has efficacy and might represent an alternative in selected settings. 

## 8. New β-Lactamase Inhibitor Combinations

Since 1966 cefepime has been employed in the treatment of *P. aeruginosa* infections due to its high potency, AmpC stability, and a chemical structure that is more protected from β-lactamases [112]. 

However, resistance to cefepime in *P. aeruginosa* is significant and is mediated by hyperproduction and stable derepression of chromosomal AmpC and/or up-regulation of efflux pumps [113,114]. 

Among cefepime-β-lactams inhibitor (BLI) combinations, which have demonstrated good in vitro activity against targeted GNB producing ESBLs, AmpC enzymes, and also carbapenemases such as cefepime-taniborbactam and cefepime-zidebactam, seem to potentiate cefepime activity against *P. aeruginosa* [115].

### 8.1. Cefepime-Taniborbactam

Taniborbactam, formerly VNRX-5133, belongs to the boronic acid BLI class, similarly to vaborbactam. Unlike DBOs, boronic acid BLIs lack intrinsic β-lactam activity. It is the first BLI with direct inhibitory activity against Ambler class A, B, C, and D enzymes. Thus, it competitively inhibits all MBLs except for IMP-type MBLs [116,117].

Unfortunately, compared with *Enterobacterales*, higher concentrations of taniborbactam were required to significantly potentiate cefepime activity against *P. aeruginosa* in an in vitro study based on ESBL isolates [118].

Recently, one Spanish study revealed that among meropenem-resistant *Pseudomonas* spp. isolates, the activity of cefepime-taniborbactam against serine-β-lactamase was comparable to that of ceftazidime-avibactam and superior to other compounds (meropenem-vaborbactam, imipenem-cilastatin-relebactam, ceftolozane-tazobactam) and superior against MBL producers compared with the same comparators, including ceftazidime-avibactam [119].

In another in vitro study taniborbactam reduced cefepime MICs by a median of five two-fold dilutions to ≤16 mg/L in 86% of MBL-producing *P. aeruginosa* [120]. 

Considering the clinical data, four phase 1 studies have assessed cefepime-taniborbactam PK in healthy volunteers and patients with renal impairment [121,122,123,124].

To date, one phase 3 non-inferiority study comparing cefepime-taniborbactam with meropenem for the treatment of cUTI due to GNB including *P. aeruginosa* and assessing the clinical efficacy of both intermittent (over 30 min) and extended-infusion (over 2 h) dosing is currently ongoing [125]. 

Furthermore, a study on a human-simulated exposure of cefepime-taniborbactam in the neutropenic murine complicated kidney infection model has demonstrated in vivo efficacy in reducing bacterial burden among all *P. aeruginosa* isolates [126].

Taken all together, these microbiological results suggest cefepime-taniborbactam as a potential future therapeutic option in patients infected with carbapenemase-producing *Enterobacterales* and CRPA isolates, including MBL producers. It is possible that an optimized drug exposure of cefepime at high doses as a prolonged infusion in combination with taniborbactam could cover most MBLs [119,120].

### 8.2. Cefepime-Zidebactam

Cefepime combined with zidebactam, which is a novel β-lactam enhancer antibiotic possessing a non-β-lactam bicycloacyl hydrazide pharmacophore, has good activity in vitro against MDR-*P. aeruginosa* due to the combination of the PBP3 inhibitor cefepime, and a PBP2 inhibitor zidebactam resulting in an enhanced bactericidal effect. The effectiveness of this β-lactam enhancer mechanism is not impacted by the concurrent expression of ESBLs, class C, OXA-48-like, and MBL-carbapenemases, despite the fact that zidebactam is a non-inhibitor of the latter two enzymes [127]. 

Past in vitro and in vivo works have established cefepime-zidebactam’s novel mechanism of action-driven coverage of MDR *Enterobacterales*, Pseudomonas, and Acinetobacter [128,129,130,131,132].

In a recent in vitro study, cefepime-zidebactam seemed to retain activity even against the most highly raised efflux group of *P. aeruginosa* isolates. Further, among 103 *P. aeruginosa* with ESBLs or MBLs, 97 (94.5%) were inhibited by cefepime-zidebactam 8 + 8 mg/L, whereas fewer than 15% were susceptible to any comparator [133]. Similar results are reported in other countries in in vitro studies [134]. 

In another Greek study focused on XDR phenotypes of GNB including *P. aeruginosa*, cefepime-zidebactam seems to retain high efficacy. This effect could be attributable to the β-lactam enhancer mechanism of action of zidebactam, which relies on its unique PBP2 binding action [127].

The PK/PD breakpoint for cefepime-zidebactam was identified based on pharmacodynamic targets derived from a neutropenic mouse infection model, and the probability of attainment targets was identified (for cefepime-zidebactam 2 g + 1 g, 1 h infusion, q8h) employing a population PK model built using phase 1 PK data [135].

Recently, a phase 3, randomized, double-blind, multicenter, comparative study has been registered in order to determine the efficacy and safety of cefepime 2 g-zidebactam 1 g intravenous (dose regimen: q8h, 1 h infusion) vs. meropenem in the treatment of cUTIs or acute pyelonephritis in adults due to GNB, including *P. aeruginosa* [133,136,137,138].

Although resistance in vitro has been already reported in some studies, this resistance seems to be the consequence of multiple mutations in genes encoding MexAB-OprM and its regulators, as well as PBP2 and PBP3. These mutations seem to negatively affect the fitness cost for cefepime-zidebactam-resistant mutants [139,140]. 

Unlike recently approved BL-BLI combinations with some gaps in the antimicrobial spectrum, cefepime-zidebactam would be least impacted by diversity in local resistance mechanisms. Thus, it could become an interesting option for those contexts (i.e., Greece, Italy, and India) where the MBL and OXA-48-like carbapenemases are concerning.

### 8.3. Meropenem-Nacubactam

Meropenem-nacubactam is a combination in early clinical development of meropenem plus nacubactam, a BLI that, like relebactam, belongs to the diazabicyclooctane (DBO) type and is structurally related to avibactam. Nacubactam has some intrinsic antibacterial activity due to PBP2 inhibition [141]. It seems to have high in vivo activity against AmpC-overproducing and *Klebsiella pneumoniae* carbapenemase (KPC)-expressing *Pseudomonas aeruginosa* isolates, without activity against MBLs [142,143].

As already mentioned, resistance mechanisms other than β-lactamase production, are not influenced by BLIs. Consequently, this combination could be more successful for CRE than for MDR and XDR *P. aeruginosa* as a future option.

## 9. Plazomicin

Plazomicin is a parenteral aminoglycoside recently approved by the FDA for the management of cUTIs and pyelonephritis caused by susceptible organisms. It has preserved activity against *Enterobacterales* producing Class A (ESBLs, KPC-2, and KPC-3), C (AmpC) and, D (OXA-48) β-lactamases. It is also active against some clinically relevant aminoglycoside modifying-enzymes (AME) and Class B β-lactamases (MBL) producers that are not co-producer of 16S rRNA methyltransferases and may have reduced activity against *Enterobacterales* that overexpress certain efflux pumps (e.g., acrAB-tolC) or have lower expression of porins (e.g., ompF or ompK36) [144].

Unfortunately, it does not seem to be superior to the other aminoglycosides amikacin, gentamicin, and tobramycin against *P. aeruginosa* and *Acinetobacter baumannii* [145].

## 10. Fosfomycin: Combination Strategy

*P. aeruginosa* contains many substrate-specific channels in its outer membrane, such as porins OprP and OprO, which are phosphate- and pyrophosphate-selective. Therefore fosfomycin as a phosphonic acid drug, with its stronger binding affinity, could be a suitable contender for permeating these porins to gain entry into *P. aeruginosa* bacteria, in particular in resistant strains [146].

The benefit of combination therapy for MDR/XDR *P. aeruginosa* infections remains controversial and the efficacy of antibiotic regimens has been investigated in in vitro studies.

Ceftolozane-tazobactam/fosfomycin dual therapy was reported to be synergic over MDR *P. aeruginosa* in a time-kill analysis [147]. With regard to ceftolozane-tazobactam in the context of CRPA strains due to MBL-production in which no activity is expected, potential in vitro synergy of ceftolozane-tazobactam in combination with fosfomycin leading to ceftolozane-tazobactam MIC reduction, represents a potential therapeutic strategy in cases of elevated MICs for both drugs in very limited options settings [148].

Considering the β-lactamase inhibitor activity of avibactam against Pseudomonas cephalosporinase and class A carbapenemases, the efficacy and synergy of the ceftazidime-avibactam/fosfomycin combination as a strategy against MDR *P. aeruginosa* has been analyzed in vitro in a high-bacterial-burden infection model. This combination led to a significant decrease in colony-forming units of *P. aeruginosa*, amounting to approximately 2 log. The combination emerged as superior compared to both drugs singly and is, therefore, a viable alternative in MDR *P. aeruginosa* isolates that are MBL negative [149].

In the context of the emergence of resistance to newer compounds such as ceftazidime-avibactam, the potential synergy of combining ceftazidime-avibactam was evaluated in a small cohort of Gram-negative, half of which were MDR *P. aeruginosa*, yielding a reduction in ceftazidime-avibactam MIC for most *P. aeruginosa* isolates in most analyzed combinations, and notably, in the combination with fosfomycin, a reduction in ceftazidime-avibactam MIC was observed in 61.9% of strains, showing the potential benefit of the combination [150].

In isolates of CRPA, analysis of the combination of fosfomycin with non-susceptible empirical antibiotics yielded in vitro synergy data in more than a quarter of all fosfomycin-antibiotic combinations tested. Susceptibility restoration was seen mostly in cephalosporin/β-lactamase inhibitor combinations, particularly in 71.4% and 68.8% of fosfomycin/ceftolozane-tazobactam and fosfomycin/ceftazidime-avibactam combinations, respectively [151].

Based on this previous evidence and susceptibility profile, algorithms for the targeted treatment of MDR/XDR ventilator-associated *P. aeruginosa* infections with recommendations for treatment selection and optimizing dosage have been proposed, placing in therapy fosfomycin combination therapy as a suitable option in case of MDR *P. aeruginosa*, particularly in the context of carbapenemase-producing MBL negative *P. aeruginosa* isolates [152].

In conclusion, due to its in vitro bactericidal activity and selectivity of membrane channels, a combined strategy with fosfomycin for the treatment of DTR *P. aeruginosa* infections has arisen. The reported data confer on old fosfomycin a new role as an intravenous formulation, emerging as a well-tolerated antimicrobial option in combination in the complex setting of MDR/XDR *P. aeruginosa* infections. A recent indication formulates intermediate daily dosing as the optimal dosage of fosfomycin in combination therapy in this selected context [152].

Besides fosfomycin, very little information about other combination treatments is available. An analysis of ceftolozane-tazobactam with various anti-pseudomonal agents against MDR *P. aeruginosa* isolates, revealed a synergy in its combination with colistin, and also in a triple regimen with fosfomycin [147]. A synergy of ceftolozane-tazobactam with aztreonam with MIC decrease in some MDR *P. aeruginosa* isolates has also been described [148]. The synergy of ceftazidime-avibactam with amikacin, aztreonam, colistin, and meropenem has been analyzed in an MDR Gram-negative small cohort, of which half were MDR *P. aeruginosa*, reporting at least a 2-fold ceftazidime-avibactam MIC reduction in almost all MDR *P. aeruginosa* isolates in most analyzed combinations [150].

## 11. Treatment Strategies

**Figure 3:**
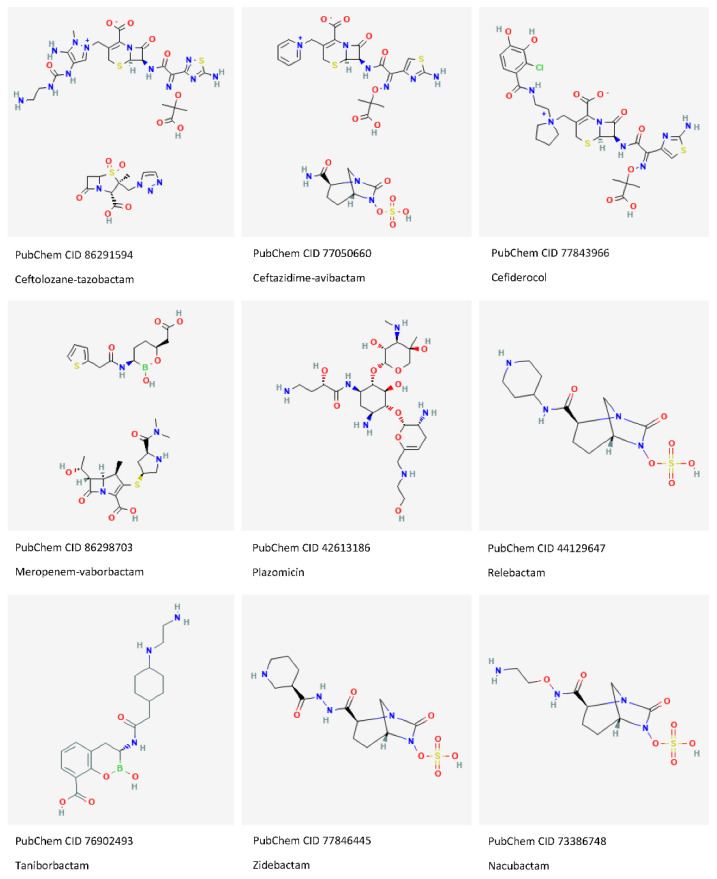
Chemical structures of anti-pseudomonal agents in use and of new β-lactamase inhibitors (Images source: PubChem, https://pubchem.ncbi.nlm.nih.gov/, “2D-Structure”, accessed on 20 April 2022).

In 2017, the WHO reported CRPA as one of the pathogens in the “critical priority” group for which new antibiotics are urgently required, but there is a visible mismatch between the newly approved antibiotics for CRE and CRPA, which are both in the WHO priority pathogens list (Figure 3). Despite the availability of new drugs in the armamentarium, also against some *P. aeruginosa* isolates with complex resistance profiles (Table 1), the development of new targeted strategies when limited treatment options are available is still needed.

According to the IDSA Guidance, ceftolozane-tazobactam, ceftazidime-avibactam, and imipenem-cilastatin-relebactam as monotherapy are the preferred treatment options for the treatment of infections outside of the urinary tract caused by DTR-*P. aeruginosa*. The main difference with the treatment suggested for UTIs is that in uncomplicated cystitis, a single dose of an aminoglycoside can be an option, whereas in cUTIs the novel agent cefiderocol can be considered [153].

ESCMID guidelines suggest ceftolozane-tazobactam (if active in vitro) as the first choice for severe infections due to CRPA, but also underline that, under the consideration of antibiotic stewardship and on an individual basis, patients with non-severe or low-risk CRPA infections, can be treated with old antibiotics, chosen according to antimicrobial susceptibility and the source of infection [37].

One of the most controversial management questions involves the use of combinations or monotherapy for serious infections due to *P. aeruginosa* and high-quality data informing the decision is still lacking, particularly in terms of reduction in mortality.

In patients with a high risk of resistant strains, in empiric therapy the potential benefits of a combination rely on the increased likelihood that at least one agent of the two is active, the likely additive or synergistic antibacterial activity, and the decreased risk of selection of a resistant subpopulation, especially when the microbial burden is high. Some data, including the last guidelines, report no value to continue combination therapy once in vitro susceptibility is confirmed [34,154].

The possible emergence of resistance to ceftolozane-tazobactam and ceftazidime-avibactam, due to higher dependence on these compounds at present, could lead to the wider use of new drug combinations to avoid the more toxic therapy with colistin.

As mentioned above, some recent in vitro studies underline the role that some old drugs, such as fosfomycin or aztreonam, could have in restoring the antimicrobial susceptibility of other “backbone” β-lactam drugs like ceftazidime-avibactam and ceftolozane-tazobactam, suggesting that, for example in cases of higher MICs, combinations with old drugs could be advantageous, especially in the contest of deep-seated, difficult to treat infections [148,149,150,155]. Despite its lack of microbiologic susceptibility, recent observations on a possible role of azithromycin against *P. aeruginosa*, even among XDR isolates, have emerged in small clinical experiences, requiring further confirmation [156].

Further, real-world evidence regarding mainly novel β-lactams such as ceftolozane-tazobactam and ceftazidime-avibactam, seems to support optimizing dosage (Table 2) as well as the administration by continuous or prolonged infusion in scenarios in which an aggressive PK/PD target is difficult to achieve, such as augmented renal clearance or deep-seated infections [46].

## 12. Conclusions

The Public Health implications of *P. aeruginosa* with limited treatment options should not be ignored as it remains one of the major causes of healthcare-associated infection in Europe.

Ceftolozane-tazobactam and ceftazidime-avibactam are safe, efficient, and carbapenem sparing options against DTR strains, but resistance against both compounds is already emerging, suggesting that, without a proper antimicrobial stewardship approach, these new drugs will lose their efficacy in a short time.

Cefiderocol could represent an option when more complex mechanisms of resistance interact together as in XDR phenotypes and MBL-producer strains, and some of the new antimicrobial combinations in the pipeline seem promising as they could also be stable against most carbapenemases.

Imipenem-cilastatin-relebactam could be considered a reasonable treatment option against emerging ceftolozane-tazobactam-nonsusceptible isolates, but real-life studies to define its role are needed.

The use of combination regimens should be assessed on an individual patient basis. Combination therapy with old drugs remains an option in case of deep-seated infections and in selected settings such as high-inoculum infections where the emergence of resistance is concerning and when MICs are high.

Knowledge of specific resistance mechanisms gained through multiplex polymerase chain reaction (PCR) platforms is crucial for the stewardship of antimicrobial weapons.

## Figures and Tables

**Table 1:** Main resistance mechanisms of new antibiotics.

Anti-Pseudomonals in Clinical Use	Main Resistance Mechanisms
Ceftolozane-tazobactam	AmpC structural mutations, β-lactam target modification (PBP) [21,22,47], OprD mutation and efflux pumps upregulation [28], MBL productions [27], OXA-2 and OXA-10 mutations [23,24,25]
Ceftazidime-avibactam	OprD mutation and efflux pumps upregulation [28,47,62,63,64], AmpC structural mutations, β-lactam target modification (PBP) [22,28,47], OXA-2 and OXA-10 mutations [24,25,65], MBL production [61]
Cefiderocol	Mutations in major iron transport pathways, possible AmpC mutations [79] mutations in β-lactamases [78]
Meropenem-vaborbactam	Porin mutations, efflux pump upregulation, MBL and OXA production [108]
Imipenem-cilastatin-relebactam	MBL and GES carbapenemases [85], mutations in MexB or in ParS [98]
Plazomicin	16S rRNA methyltransferases (i.e. Rmt or Arm) [145]

**Table 2:** Clinical dosage and renal adjustment for anti-pseudomonal agents.

Drug	Clinical Dosage	Comments
Ceftolozane-tazobactam	1.5 g (ceftolozane 1 g/tazobactam 0.5 g) intravenous every 8 h over 1 h 3 g (ceftolozane 2 g/tazobactam 1 g) intravenous every 8 h over 1 h for HAP/VAP	Extended infusion (over 3 h) 1.5 g or 3 g every 8 h is recommended [46] Renal adjustment with CrCl < 50 mL/min
Ceftazidime-avibactam	2.5 g (ceftazidime 2 g/avibactam 0.5 g) intravenous every 8 h over 2 h	Extended infusion (over 3 h) 2.5 g every 8 h is recommended [46] Renal adjustment with CrCl < 50 mL/min
Cefiderocol	2 g intravenous every 8 h over 3 h	Renal adjustment with CrCl < 60 mL/min
Imipenem-cilastatin-relebactam	1.25 g (imipenem 500 mg/cilastatin 500 mg/relebactam 250 mg) intravenous every 6 h over 30 min	Renal adjustment with CrCl < 90 mL/min
Meropenem-vaborbactam	4 g (meropenem 2 g/vaborbactam 2 g) intravenous every 8 h over 3 h	Renal adjustment with CrCl < 50 mL/min
Plazomicin	15 mg/kg every 24 h over 30 min	Renal adjustment with CrCl < 60 mL/min
Fosfomycin	6–8 g loading dose intravenous, followed by 16 g/day [152]	Renal adjustment with CrCl < 40 mL/min
